# Progressive supranuclear palsy: diagnosis and management

**DOI:** 10.1136/practneurol-2020-002794

**Published:** 2021-07-02

**Authors:** James B Rowe, Negin Holland, Timothy Rittman

**Affiliations:** 1 Department of Clinical Neurosciences, University of Cambridge, Cambridge, Cambridgeshire, UK; 2 MRC Cognition and Brain Sciences Unit, Cambridge, Cambridgeshire, UK; 3 Cambridge University Hospitals NHS Foundation Trust, Cambridge, Cambridgeshire, UK

**Keywords:** movement disorders, dementia, cognition, supranuclear palsy

## Abstract

Treating patients with progressive supranuclear palsy (PSP) is both effective and rewarding. This review aims to share our experience in the proactive management of PSP, considering the patient, the family and the medical context in which the illness unfolds. There are many opportunities to assist your patients, ameliorate their symptoms, reduce their risks and harm, and guide them through the complex medical, social and legal minefield that characterises life with chronic neurological illness. We summarise the challenges of early diagnosis, consider PSP mimics and the role of investigations in excluding these, and discuss the available pharmacological and non-pharmacological treatment strategies to tackle the common and challenging symptoms of PSP. The best treatment will be patient centred and as part of a multidisciplinary team.

## Introduction

Progressive supranuclear palsy (PSP) is not yet curable, but many aspects are certainly treatable. Indeed, the support and management of people with PSP can be both effective and rewarding. This article shares our experience in its proactive management, considering the patient, the family and the medical context in which the illness unfolds. Effective management starts with achieving an early, accurate diagnosis. We review new concepts in the diagnosis of PSP that have emerged over the last 10 years. We then consider the differential diagnosis and the role of investigations. We go on to review pharmacological treatment options and non-pharmacological aspects to effective holistic care, support and management.

### Diagnosis of PSP

By the time of diagnosis, patients with PSP are typically 3 years from their first symptom.^1^ That is halfway through the illness. The lateness of diagnosis is multifactorial with delays seeking general practitioner advice, failure to recognise the significance of early symptoms and misdiagnosis as depression and/or Parkinson’s disease. Part of the problem is the terminology of ‘atypical Parkinsonism’. There is nothing ‘atypical’ about PSP: it is typical of PSP, and readily distinguished from Parkinson’s disease. For example, the limb signs in PSP are symmetrical, without tremor, and rigidity is marked in the trunk and neck, and minimal in the periphery—the opposite of Parkinson’s disease on all counts.

The diagnosis of PSP is usually not difficult with a few easy tips, set out in table 1. Many of these apply even soon after symptom onset. Is there a tendency to fall over easily, in the first year of symptoms? Yes for PSP, no for Parkinson’s disease. Are the eyes ‘staring’, with an odd, fixed smile or grimace? Yes for PSP, no for Parkinson’s disease. Do they walk with head up and forward (poetically called sniffing the morning breeze)? These features take seconds to take in as the patient walks to the consulting room.

**Table 1:** Symptoms and signs of PSP

Symptoms	Signs
Cognitive change (apathy, impulsivity)	Akinetic rigidity—neck and axial rigidity >limbs ‘sniffing morning breeze sign’
Impaired balance	Slow saccades and ‘round the houses’ vertical saccades
Early falls (with increased fracture risk)	Vertical supranuclear gaze palsy
Blurry or double vision	Frontalis overactivity, reduced blink, staring expression
Sleep difficulties	Tendency to lose balance spontaneously or on the ‘pull test’
Dysphagia (especially liquids)	Uncontrolled decent into a chair
Drooling, sialorrhoea	Dystonia, cervical, axial >limbs
Urinary urgency or incontinence	Apraxia (CBS overlap)
Constipation	Emotional lability (pseudobulbar affect)
Depression or anxiety	Reduced verbal fluency
Hyperphagia and change in food preferences	Dysarthrophonia
Weight loss (with possible malnutrition)	

CBS, corticobasal syndrome; PSP, progressive supranuclear palsy.

Once in the consulting room, further differences are obvious. Typical PSP speech is not the quiet, hypophonia of Parkinson’s disease, but a more chaotic dysarthrophonia: distorted, slow and effortful, sometimes inappropriately loud then indistinct, often nasal in quality or low pitched with a ‘gravelly drawl’. Speech may be mixed in with laughter, perhaps inappropriately. There is much less spontaneous speech—known as adynamic aphasia.

There may be changes in personality. In the history, families may have noticed the person with PSP becoming apathetic (losing ‘get up and go’, zest, motivation, or interest); or becoming self-centred and stubborn. These reflect the common cognitive impairments of PSP, and its relationship to frontotemporal dementias.^2^ Despite a history of apathy, patients may also be impulsive, leaping up from their chair as you approach, or in anticipation of your request to stand, even if balance is poor.

**Figure 1:**
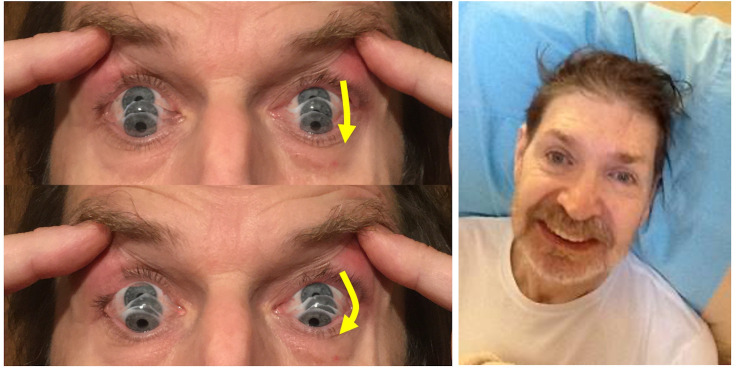
Left: ‘Round-the-houses’ sign, illustrated for a downward saccade. Note the lateral curvature of downward path of eye movement (yellow arrows). The velocity may also be slow. Right: the face gives many clues to the diagnosis: as shown by this gentleman, there may be retrocollis, raised eyebrows and frontalis overactivity. People with PSP may have a rather fixed smile, with lips drawn back rather than up. The eyebrows sometimes appear knitted together (not shown). For video clips of other signs of PSP, refer to the ‘quick links to further information section’. PSP, progressive supranuclear palsy.

Look at their eyes. You may notice a blank, staring appearance, with few spontaneous blinks, a furrowed brow and raised eyebrows. Look closely for small square wave jerks in neutral gaze. These are small (2°–4°), horizontal, brief (less than a second before returning to target), and frequent (every few seconds). Early in the course of PSP, there can be a full range of eye movements, but a key feature for the diagnosis is the presence of either (1) restricted vertical saccadic movements in a supranuclear pattern or (2) slow saccades (tip: compare vertical to horizontal saccades). A valuable sign that is not in the formal diagnostic criteria, but which can be seen before restriction and slowing, is a curvilinear path on downward saccades (‘round-the-houses’ sign, see figure 1). Slowing and ‘round-the-houses’ are best seen with saccadic movements on command to a target. The supranuclear gaze palsy from which PSP is named causes restriction of voluntary eye movements in the vertical plane (up, down or both). The patient overcomes the restriction (at least in part) by reflexive eye movements when you, the clinician, tilt the patient’s head with them fixating on a target (Tip: if the neck becomes too rigid to tilt the head, try holding gently with both hands either side, and wobbling it a few degrees left and right first, before a downward tilt). Other dementia syndromes can cause a ‘pseudo-gaze palsy’ due to ocular apraxia (eg, posterior cortical atrophy, Alzheimer’s disease and corticobasal degeneration, Huntington’s disease), in which a patient initially appears unable to look up or down to a target, while reflexive movements are preserved with head tilting. However, with repeated attempts, it is possible in these conditions to elicit a fast and full saccade to the vertical target.

Are they dopa-responsive or not dopa-responsive? One of the criteria for the diagnosis of PSP is that it is poorly responsive to levodopa. This is poor with respect to the level of response in Parkinson’s disease. But ~30% of patients report some benefit to levodopa or dopamine agonists. The effect is mild, and they may describe vague benefits in mobility, speech, ‘energy’ and other symptoms. Only conclude non-responsiveness if a high dose has been given (750–1000 mg daily). Note that PSP does not cause dyskinesia, or dopa-induced dyskinesia (unlike Parkinson’s disease). The dose can be rapidly increased over 6–8 weeks and decreased rapidly if not effective.

So, PSP is not Parkinson’s disease! It is usually not difficult to diagnose the classical presentation, known commonly as Richardson’s syndrome. Table 2 gives a list of distinguishing features, but just ask yourself, ‘does this person actually look like the other people with Parkinson’s disease that I have seen?’ The answer, with PSP, will be ‘No’.

**Table 2:** Key differences in symptoms and signs in PSP and Parkinson’s disease

	PSP	Parkinson’s disease
Symmetrical	**Yes**	**No**
Rigidity	**Axial**	**Limb**
Akinesia	Severe, global Even in loose limbs	Mild to moderate
Tremor	**No**	**Yes**
Falls	**Early, spontaneous**	**Late, with freezing**
Eyes	**Vertical paresis**	Normal*
Voice	Dysarthrophonia, distorted, poor volume control	Hypophonia, quiet
Cognition	Marked early executive changes Loss of fluency	Subtle early executive changes or later dementia
Levodopa	Poor response	Very good response
Gait	Head up, sniffing the air Leaning back	Head down, stooped, leaning forward
Looks like Parkinson’s?	**No**	**Yes**

Bold text highlights the simplest quick-six to have in mind.

* Subtle oculomotor abnormalities occur

PSP, progressive supranuclear palsy.

### New variants of PSP

When Steele *et al*,^3^ first described PSP, seven of their nine cases had dementia or severe cognitive and behavioural change. Despite this, the illness became known as a movement disorder, allied to Parkinson’s disease—a problem exacerbated by terms like ‘Parkinson-plus’ and ‘atypical parkinsonism’. However, the importance of cognitive and behavioural change has increased with recent recognition that cognitive change is almost universal in PSP, and that up to half of patients do not present with the classical Richardson’s syndrome.

The new 2017 diagnostic criteria for PSP^4^ recognise these ‘variant’ presentations. Most eventually evolve to look like classical Richardson’s syndrome, but knowing the variants will allow you to make an earlier diagnosis with confidence. They include:

1. PSP-Speech and Language (SL) with a non-fluent aphasia, preceding typical changes of PSP.
2. PSP-Frontal (F): prominent apathy, impulsivity and inappropriate behaviour.
3. PSP-corticobasal syndrome (CBS) with CBS-like signs of asymmetric dystonia, apraxia, cortical sensory loss, myoclonus or alien-limb.
4. PSP-Progressive gait freezing (PGF), with sudden motor block, hesitation or initiation failure when walking. PSP-PGF can occur many years before oculomotor signs and is highly predictive of PSP. Akinesia is profound, while cognition remains essentially intact.
5. PSP-Parkinsonism in which patients begin with asymmetry in limb features, tremor, and even levodopa responsivity, later developing more typical PSP features (Richardson’s syndrome).

The new criteria also introduce formal levels of diagnostic certainty, from definite (pathology confirmation); probable, possible and ‘suggestive of’ PSP. The latter class of ‘suggestive of’ PSP refers to people with limited signs of PSP, failing to meet former standard criteria, but nonetheless with a significant prognostic value for PSP. In this case, be honest about your suspicion of PSP, initiate symptomatic treatment and follow-up.

These new categories of PSP may seem complex. But, the details matter less than recognition that PSP presents with a wide range of changes in cognition, language and behaviour.

### Differential diagnosis and the [limited] role of investigations

PSP is essentially a clinical diagnosis, and when made by a neurologist has very high clinicopathological correlations. There are differential diagnoses (table 3), with clinical clues to guide you. Investigations are mainly used to rule out mimics and to look for uncommon reversible alternatives. We present them in a didactic manner, but this is no substitute for clinical judgement of a given case.

**Table 3:** Differential diagnosis for progressive supranuclear palsy other than Parkinson's disease.

Disease	Clues to diagnosis
CBS	Early asymmetric akinesia, apraxia, dystonia, myoclonus
MSA	Predominant autonomic features, cerebellar signs
FTD	Predominant behaviour features, marked atrophy
NPH	Supportive imaging findings
Vascular disease	Supportive imaging findings, vascular risk factors
Structural lesion	Supportive imaging findings
Rare genetic mimics	Young age of symptom onset, relevant family history
DLB	Hallucinations and fluctuations common
AD	Disproportionate memory impairment, hippocampal atrophy on MRI, suggestive CSF biomarkers

These differential diagnoses may cause abnormal eye signs, but lack the distinctive selective vertical gaze palsy and slowing of PSP and the other characteristic clinical face and limb signs of PSP.

AD, Alzheimer's disease; CBS, corticobasal syndrome; DLB, Dementia with Lewy bodies; FTD, Frontotemporal dementia; MSA, Multiple System Atrophy; NPH, Normal Pressure Hydrocephalus; PSP, progressive supranuclear palsy.

Blood tests may occasionally identify unlikely mimics (low thyroid stimulating hormone, positive syphilis serology), but are more useful to consider the consequences of disability and guide falls risk-reduction (full blood count, clotting, bone profile, vitamin D, B_12_, folate), or to manage latent comorbidities that complicate the course of PSP (glycated haemoglobin HbA1c, urea, creatinine and electrolytes). More exotic tests (HIV, copper, caeruloplasmin, ferritin, antinuclear antibody, genetics, white cell enzymes) may be indicated in young onset cases (<50 years) or atypical phenotypes (movement disorders other than those above or with white matter pathology on MR brain scan).

PSP is a sporadic disease, and routine genetic testing is not indicated. However, if there are first degree relatives with a neurodegenerative condition under 65 years, or multiple relatives with neurodegenerative disorders, then consider genetics testing with C9orf72 and a next-generation sequencing dementia panel. Most relevant disorders in the family are PSP, Parkinson’s disease (try to get an account of the relative’s symptoms), frontotemporal dementia, progressive aphasia, motor neurone disease. Disorders that broadly resemble PSP can arise from mutations in C9orf72 (overlapping with frontotemporal dementia, motor neurone disease, aphasia, ataxia), microtubule associated protein tau (overlapping frontotemporal dementia, CBS), progranulin (GRN, overlapping frontotemporal dementia, aphasia), Park9 (Kufor-Rakeb syndrome) and CSF-1R (hereditary leukoencephalopathy with axonal spheroids) among others. Niemann-Pick type C is often considered as a differential diagnosis, although the gaze palsy is horizontal or global, not selectively vertical.

**Figure 2:**
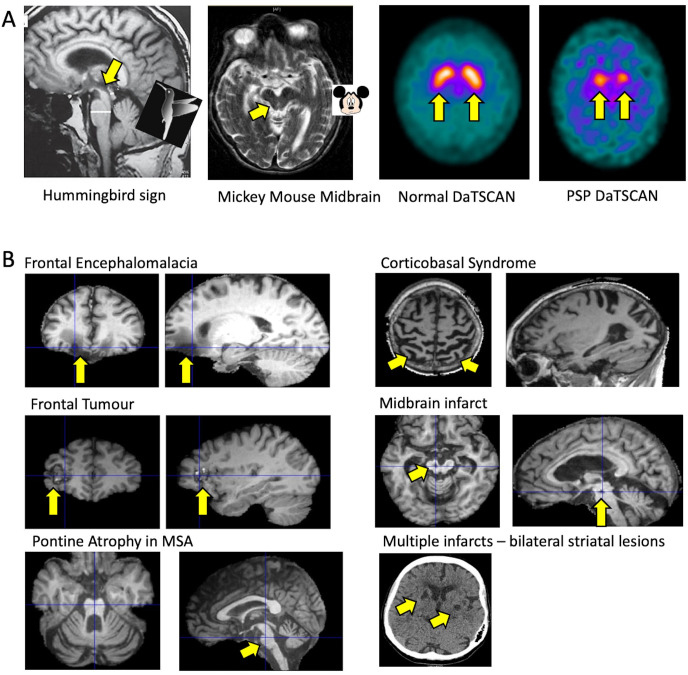
MRI and DaTscan features in PSP (A) and examples of MRI features of alternative diagnoses for PSP-like presentations (B), with arrows highlighling the salient feature or anomaly. MSA, multiple system atrophy; PSP, progressive supranuclear palsy.

MR scan of brain is the most useful diagnostic tool regarding PSP, not so much for the menagerie of positive signs (hummingbird sign, Mickey Mouse sign, figure 2A), but for its ability to exclude alternative diagnoses such as extensive small vessel disease, leukodystrophy, normal pressure hydrocephalus and frontal mass lesions (figure 2B).^5^

A DaTSCAN is rarely indicated. It will be abnormal in PSP (figure 2A), but it will also be abnormal in Parkinson’s disease, multiple system atrophy, and usually with corticobasal degeneration and frontotemporal dementia with parkinsonism. As such, the DAT scan is not helpful for differential diagnosis. It is rare that drug-induced parkinsonism or essential tremor are serious differentials for PSP. DaTSCANs add little to MR brain scan in differentiation of degenerative parkinsonism from vascular disease or normal pressure hydrocephalus (although in the latter two it is usually normal). One area when a DaTSCAN can help is in distinguishing PSP from primary lateral sclerosis variant of motor neurone disease, which can cause akinesia (although without decrement), oculomotor slowing (including horizontal) and dysarthria. Fluorodeoxyglucose positron emission tomography (FDG-PET) and ‘tau-PET’ are abnormal in PSP but are still primarily research tools.

### Drug treatment of PSP

PSP cannot yet be cured, but treatment can be helpful. Each patient is different, and so the selection, timing and doses of the following drugs will vary from person to person. Except for dopaminergic therapy, the mantra should be ‘start low and go slow’, as PSP increases sensitivity to side effects, which can have severe consequences in a fragile medical condition or precarious social situation.

### Symptom reduction

For many cognitive and behavioural symptoms, support, tolerance and environmental measures are more effective—and safer—than medication. Experience (anecdote) and cohort studies dominate the evidence base, over randomised, controlled clinical trials. So, focus on medications that aim to reduce those symptoms that trouble the patient, or place them at risk. The following sections are a guide, not a substitute for clinical judgement. Ask the patient which of all the problems caused by PSP actually bothers them—the answers may surprise you! PSP robs people of so many abilities and aspirations, but professionals often unwittingly remove the patients’ autonomy. Patient-centred treatment is more satisfying, more likely to succeed and better for risk-benefit decisions.

Motor symptoms: We recommend a trial of levodopa in all patients with significant akinetic rigidity, but usually not in people with PSP-F or PSP-SL. The range of options is the same as in Parkinson’s disease, but prescribing practice differs in PSP because of (1) the need for early clarity over responsiveness; (2) the faster rate of progression of PSP and (3) the much lower rates of common side effects of levodopa in PSP for example, less likely to cause nausea, hypotension and dyskinesia (reflecting different striatal and autonomic pathology). We recommend 62.5 mg three times a day, doubling after 2 weeks and again after 2 weeks, reaching 250 mg three times a day by 1 month, with further increases to 1000 mg/day if tolerated. This is much faster than one would escalate in Parkinson’s disease. We assess response by the patient-based and carer-based global impression of change by 2–3 months. If it is not noticeably of benefit, then withdraw over 4–6 weeks. Dopamine agonists are a reasonable alternative to levodopa. They may be of particular value in PSP-PGF, using high doses (eg, increasing rotigotine stepwise to 16 mg/day).

Amantadine can be helpful, with patients reporting reduced axial rigidity, improved mobility, more ‘energy’ and less fatigue, or clearer speech. Younger patients, aged under 60, are more likely to report benefits, while older patients, aged over 75, are more likely to experience significant side effects, for example, hallucinations and oedema. Start low (100 mg at breakfast) and increase by 100 mg at two weekly intervals aiming for 300–400 mg total dose. Ensure that the second dose is not after lunchtime, as it may cause insomnia.

PSP-PGF may respond to cholinesterase inhibitors, reflecting the cholinergic nature of pedunculopontine gait-rhythm generators. With the generally favourable safety profile of these drugs, it is worth considering a trial period of donepezil (starting 5 mg, rising steadily to 20 mg once daily).

Myoclonus is rare in PSP but can occur in PSP-CBS. It rarely troubles the patient, even if obvious to others. It often responds low dose clonazepam (0.25–0.5 mg, once a day to two times a day), levetiracetam or lamotrigine, but avoid valproate in view of its potential to exacerbate parkinsonism.

Dystonia can be severe, and painful, with cervical dystonia affecting swallowing and social interaction. Topical non-steroidal anti-inflammatory drugs are a good first line treatment, particularly for shoulder dystonia. Amantadine may help but is unlikely to be sufficient on its own. Cholinergic approaches are not advised, because of their deleterious effect on cognition, balance and falls risk. Botulinum toxin is most effective, especially if combined with physiotherapy.

For bladder symptoms (eg, urgency and incontinence), we recommend excluding other causes (eg, prostate enlargement, diuretics, caffeine) and using mirabegron. This has an advantageous side-effect profile over anticholinergic alternatives, although monitor for hypertension.

Psychiatric features of PSP: Given the degree of disability, depression is surprisingly uncommon in PSP. Akinesia, apathy and pseudobulbar affect (below) may be confused with depression. Patients can score highly on depression rating scales (from changes in sleep, energy, motivation, libido, etc), but the core symptom of low mood is rarely severe or persistent. A minority of patients have significant and pervasive depression and anxiety. We avoid tricyclics (eg, amitriptyline) because of the anticholinergic effects. Selective serotonin reuptake inhibitors (SSRIs), especially citalopram and sertraline, are well tolerated and effective, with potential additional benefits for impulsiveness and pseudobulbar affect. Mirtazapine can be particularly useful if night-time sedation or increased appetite is desirable. Note that 15 mg mirtazapine is paradoxically more sedating and less antidepressant than 30 mg. Venlafaxine (a noradrenaline/serotonin reuptake inhibitor SNRI/SSRI) may help where there is accompanying anxiety.

People with PSP may suddenly cry intensely, appearing very distressed and tearful. This pseudobulbar affect is upsetting to witness but does not always reflect a patient’s inner distress. If asked, between the tears, they may deny feeling upset, or be aware of a surge of emotionality. The emotional lability can be laughter but is more often crying. If it is frequent, or distressing, then consider treatment; low dose citalopram (10–20 mg) is usually very effective.

Sleep disturbance is common in PSP, but often not volunteered. Simple measures may be sufficient: stopping diuretics; treating prostatic hypertrophy; recommending both increased daytime activity and exercise and a limit on daytime naps to 30 min or less; reviewing and treating of depression and anxiety; and ensuring that drugs like amantadine that can cause insomnia are reviewed and taken earlier in the day. If this is not sufficient, then primary insomnia may respond to ‘Z-drugs’ like zolpidem, melatonin or short-term benzodiazepines, balancing the risks of sedation and falls against lack of sleep.

Keening, wailing, and perpetual ‘stammer’: Some patients utter repetitive, loud vocalisations for minutes or hours on end. This is exhausting to be near, and may be the last straw for carers at home, and may even prevent care-home placement. It resembles an extreme stammer, but with a quality that might suggest distress. It helps to explain to carers that it rarely indicates distress, and that it is part of the illness. If worse at night, then sedation as above may help.

Drooling and sialorrhoea: Drooling in PSP results from not swallowing saliva, rather than overproduction. It may be exacerbated by anterocollis: repositioning may be all that is required. We advise against hyoscine patches because of the harmful anticholinergic effects. A better alternative with negligible systemic side effects is oral atropine drops, 2–3 sublingual drops, 2–3 times per day. The number of drops and frequency can be readily titrated, particularly if saliva becomes too thick. Alternatives are: salivary gland botulinum toxin, which is a less reversible/titratable option; and oral glycopyrronium, although we are not convinced of its value in PSP given that it primarily aids chest secretions and may have systemic side effects.

Involuntary eye closure is common in PSP. It can be mild and irritating or severe with functional blindness. It may be caused blepharospasm (especially pretarsal blepharospasm) or apraxia of lid opening—the inability to open the eyes voluntarily despite normal peripheral levator function. It may respond well to botulinum toxin injections. Eyelid opening apraxia (not blepharospasm) is a diagnostic criterion for PSP but is uncommon in the early stages.

### Reducing risk

Alongside the reduction of symptoms, be proactive to reduce the future risks to patients. Maintaining mobility and walking helps in terms of independence, balance and general health. However, falls are an inevitable feature of PSP, and can lead to serious injury, a premature loss of functional independence, and anxiety. We have reviewed the approach to falls management in PSP elsewhere.^6^ While there is no strong evidence-based approach to reducing falls risk in PSP, we advocate a personalised approach to address polypharmacy, nocturia and impulsivity.

Consider bone protection to reduce the risk of fracture. Vitamin D deficiency is very common in the UK, and particularly so among people with PSP. Community fracture risk estimates (eg, “FRAX” Fracture Risk Assessment Tool scores) do not accommodate the falls frequency of PSP. We therefore recommend a low threshold for DEXA scanning, and proactive management of osteopenia and osteoporosis with Calcium-D3, and a bisphosphonate where indicated. The dysphagia of PSP increases the risk of severe oesophageal injury with oral bisphosphonates, so infusion-based alternatives may be required.

Dysphagia leading to weight loss and aspiration is common in PSP. We recommend monitoring weight and advising families to come forward if there are symptoms of a chest infection. Aspiration may be heralded by coughing and choking but may be silent. Seek early assessment by speech and language therapy colleagues, with a view to advice on safer eating, timing of modified diets and fluid thickeners, and discussions in selected patients on feeding alternatives such as percutaneous endoscopic gastrostomy (PEG) tube. PEG reduces but does not entirely prevent aspiration. Exhaustingly long mealtimes, weight loss, reduced resilience to infection and pressure sores, and frightening episodes of choking may all be ameliorated by PEG feeding. A PEG tube does not exclude eating for pleasure: favourite food and drink can be a continuing source of enjoyment and social engagement, while the PEG handles the bulk nutrition. In our clinic, about half of patients accept the offer of PEG.

Arguably the most important way to reduce risks is to avoid harmful medication. People with PSP can be exquisitely sensitive to neuroleptics, with catastrophic and irreversible worsening of an extrapyramidal syndrome. Psychosis and agitation are rare, although can be induced by medications (eg, anticholinergics, opiates, amantadine) and infection (chest, urine, even without fever). Short courses of short-acting sedatives or hypnotics have a role, provided other exacerbating factors have been addressed. But avoid typical (eg, haloperidol) and most atypical (eg, risperidone) neuroleptics. Quetiapine probably carries least risk of harm, but with questionable efficacy.

Review other medications and try to reduce well-intentioned but unnecessary polypharmacy. For example, was the speech problem attributed to a small stroke actually the start of the PSP? If so, the aspirin and angiotensin converting enzyme inhibitor may not be needed. Is there a solid reason for that statin? Remember that patients with PSP have survival expectation of 3 years from diagnosis, not a 10-year modifiable cardiovascular risk. Focus on treatments that are priorities for symptoms and significant health risks.

### Disease-modifying treatments

Several agents have been tried in completed phase II/III clinical trials of Richardson’s syndrome. These have been successful in galvanising the international PSP community, and demonstrating feasibility of PSP trials, with important lessons learnt about trial design and end points. They have not shown clinical efficacy. However, several clinical trials are underway and in preparation for 2021–2022. We look forward to one of these demonstrating efficacy to slow progression. But there is no need to be passive in the interim, with so much else to consider in treating the symptoms that trouble the patient.

### Non-drug support

The coordination of non-pharmacological management is as important as drug management. Support for patients and families at different stages of disease will require input from a diverse multi-disciplinary team, from hospital and community services in health and social care. The provision of these services, and professional boundaries and titles, varies greatly between regions. These teams often look to the patient’s neurologist for information, recommendations, and advice. So, what can you do?

Be liberal with ‘to whom it may concern letters’ as these improve access to medical services in the community, social services support or even travel insurance to enable patients to take a break or meet family abroad. Send copies of your clinic letters to patients, so they have critical information to hand when meeting other doctors or are admitted to hospital elsewhere.

The multidisciplinary team and other professional partners in care may include, but are not limited to:

1. General practitioner.
2. Specialist nurses: Parkinson’s disease specialist nurse, neurology specialist nurse, dementia specialist nurses.
3. District nurse, and community matron.
4. Palliative care team, hospice and home support.
5. Physiotherapist: ideally neurophysiotherapy specialist team.
6. Occupational therapist.
7. Speech and Language therapist.
8. Feeding-issues team/gastroenterology multidisciplinary team.
9. Continence adviser.
10. Dietician.
11. Community mental health team, and community psychiatric nurse.
12. Social worker.
13. Community pharmacist.
14. Independent living team.
15. Advisers on financial support, benefits, pensions, power of attorney, from third sector organisations (eg, Citizens’ Advice Bureau, AgeUK, Alzheimer society, PSP Association).
16. Independent adviser on continuing healthcare (eg, Beacon Healthcare).

Try to identify a named keyworker to coordinate care and help the patient and carer navigate the health and social care system, with its Byzantine procedures. In direct support of your medical care, the Speech and Language therapist is especially important. Almost every person with PSP will develop dysphagia sooner or later. Early awareness and assessment are critical, with practical measures the patient and carer can take to reduce the risk of aspiration and, in conjunction with a dietician, reduce the risk of malnutrition. Recurrent aspiration accelerates the course of disease. Patients with PSP may be in a catabolic state, which, together with slow eating, leads to calorie deficits and vitamin deficiency, with weight loss that exacerbates fatigue and risks of injury from falls.

Physiotherapists and occupational therapists play a key role in PSP care, and in many areas provide a coordinated community-based service. The PSP Association publish ‘A guide to PSP and corticobasal degeneration for occupational therapists’, setting out roles and activities as educators and network facilitators, and as problem solvers for safe independent living. Mobility, transfers, falls management, personal care, vision, eating, cognitive change, palliation and goal setting for rehabilitation: these complex domains of PSP can each benefit from physiotherapy and occupational therapist assessment and support for the patient and carers.

### Palliative care

The involvement of palliative care teams can be hugely beneficial. Such teams often have a wide range of services, many not available elsewhere, and more readily accessible. These may include psychological support or group sessions. The timing of palliative care input can be a delicate discussion, but in general we advocate early involvement, particularly where anxieties about the end of the illness are a major issue. The palliative care team may help advanced care planning, symptom control, family support and day-centre activities.

### Empowered patients and expert families

PSP is rare. Most of the people your patients meet on their pathway through health and social care will not have heard of it, let alone seen it. This is a source of great frustration, and undermines confidence in the services they need. You can help the patient and family by building their experience, knowledge and confidence, to become ambassadors and experts in PSP. They can be given the confidence to take the lead and help those they meet to orientate quickly to PSP.

The PSP Association is a valuable source of information—for you, the patient, their family and other professionals. Their website https://pspassociation.org.uk/ and phoneline 0300 0110 122 are a portal for:

1. A group for newly diagnosed people.
2. Local groups, online support and blogs for more experienced patients and carers.
3. Support for children affected by PSP in the family.
4. Information and downloadable leaflets about PSP, in part to give to others they meet.
5. Interactive resource for professionals https://hscpguide.com/ and downloadable guidance for general practitioners, occupational therapists, physiotherapists, National Health Service continuing healthcare funding and more.
6. Contact for care-workers supporting people with PSP.
7. Educational days for health and social care professionals.
8. Support for carers

It is sensible for patients and families dealing with chronic progressive neurological conditions to set up Lasting Powers of Attorney, but this needs to be done while the patient has mental capacity. Mental capacity for major health and financial decisions is usually preserved in PSP, until very late in the illness. Mental capacity rests on the ability to:

1. Understand information given: in PSP, use simplified language, short direct sentences, and in a form the person can read or hear clearly.
2. Retain that information long enough to be able to make the decision: in PSP, this needs an unhurried and non-distracting environment.
3. Weigh up the information available to make the decision: PSP causes slow thinking and executive impairment. Patience is needed on your part. In normal conversations, a reply begins within half a second, but in PSP it may take 10–20 s.
4. Communicate a decision: this can be especially difficult but make it your problem not theirs. Speech may be a whisper, but that is sufficient. Communicating with a thumbs up/down is also usually sufficient to indicate autonomous decision making, however careful we need to be to understand the decision. Every effort must be made to find ways for the patient to communicate, before communication difficulty is misinterpreted as loss of mental capacity.

## Conclusion

Working with patients and families affected by PSP provides a great opportunity for active management. It begins with early diagnosis, to make sense of their experiences including the change in cognition, personality and behaviour. This leads on to proactive management of the myriad symptoms of PSP and risk mitigation, drawing on basic principles of good medical care. It is easy to bamboozle someone with PSP—but you can be their champion, to empower them through the health and social care system, to deal with their priority symptoms and to defend their rights and autonomy.

### Quick links to further information

Video examination signs: www.sciencedirect.com/science/article/pii/S1353802020302078?via%3Dihub

**Key points**

1. Progressive supranuclear palsy (PSP) is very different to Parkinson’s disease with readily distinguishable features
2. PSP is a clinical diagnosis; imaging helps to differentiate mimics.
3. Non-pharmacological management of PSP is as important as pharmacological treatment and should be implemented early.
4. Empower patients and their families to make well-informed autonomous decisions about their care, and aim to preserve their quality of life.

**Further reading**

1. Coyle-Gilchrist ITS, Dick KM, Patterson K, et al. Prevalence, characteristics, and survival of frontotemporal lobardegeneration syndromes. Neurology 2016;86:1736–43.
2. Murley AG, Coyle-Gilchrist I, Rouse MA, et al. Redefiningthe multidimensional clinical phenotypes of frontotemporal lobar degeneration syndromes. 2020;143:1555-71.
3. Steele JC, Richardson JC, Olszewski J. Progressive supranuclearpalsy. A heterogeneous degeneration involving the brainstem, basal ganglia and cerebellum with vertical gaze andpseudobulbar palsy, nuchal dystonia and dementia. Arch Neurol1964;10:333–59.
